# Cytopenia after CAR-T Cell Therapy—A Brief Review of a Complex Problem

**DOI:** 10.3390/cancers14061501

**Published:** 2022-03-15

**Authors:** Naman Sharma, Patrick M. Reagan, Jane L. Liesveld

**Affiliations:** 1 Department of Hematology-Oncology, Baystate Medical Center, University of Massachusetts Medical School, Springfield, MA 100107, USA; Naman.Sharma@baystatehealth.org; 2 Department of Medicine, Hematology-Oncology, James P. Wilmot Cancer Institute, University of Rochester, Rochester, NY 14642, USA; Patrick_Reagan@urmc.rochester.edu

**Keywords:** chimeric antigen receptor T-cell (CAR-T), prolonged cytopenia, cytokines

## Abstract

**Simple Summary:**

Chimeric Antigen Receptor T-cell (CAR-T) immunotherapy has emerged as a new life-saving treatment modality in patients with relapsed or refractory B-cell malignancies and multiple-myeloma. In this form of therapy, patient’s T-cells are modified by various genetic techniques to express a new receptor that identifies and kills the cancer cells. With increasing use, they present with distinct immune mediated side effects such as cytokine release syndrome (CRS), neurotoxicity, and prolonged cytopenia. While our understanding of CRS and other immune side-effects has increased, prolonged cytopenia post CAR-T infusion remains under-recognized and under-reported. With the focus on prolonged cytopenia in this review, we aim to summarize findings of various clinical trials, postulated mechanisms, and clinical interventions to risk-stratify and manage this clinical entity.

**Abstract:**

Chimeric Antigen Receptor T-cell (CAR-T) immunotherapy has emerged as an efficacious and life extending treatment modality with high response rates and durable remissions in patients with relapsed and refractory non-Hodgkin lymphoma (NHL), follicular lymphoma, and B-cell acute lymphoblastic leukemia (B-ALL) as well as in other diseases. Prolonged or recurrent cytopenias after CAR-T therapy have increasingly been reported at varying rates, and the pathogenesis of this complication is not yet well-understood but is likely contributed to by multiple factors. Current studies reported are primarily retrospective, heterogeneous in terms of CAR-Ts used and diseases treated, non-uniform in definitions of cytopenias and durations for end points, and vary in terms of recommended management. Prospective studies and correlative laboratory studies investigating the pathophysiology of prolonged cytopenias will enhance our understanding of this phenomenon. This review summarizes knowledge of these cytopenias to date.

## 1. Introduction

Chimeric Antigen Receptor T-cell (CAR-T) immunotherapy targeting the CD-19 B-cell receptor has emerged as an efficacious and life-extending treatment modality with high response rates and durable remissions in patients with relapsed and refractory non-Hodgkin lymphoma (NHL) [1], follicular lymphoma [2], and B-cell acute lymphoblastic leukemia (B-ALL) [3]. CAR-T cells targeting the B cell maturation antigen (BCMA) in multiple myeloma (MM) are now also approved for clinical use. Investigational CAR T-cells targeting the CD30 antigen in T-cell and Hodgkin lymphomas, as well as CAR-T cells directed to multiple other tumor types are also being utilized.

The process of making CAR-T cell constructs involves isolating the patient’s T-cells or T-cells from an allogeneic donor source and using lentiviral or retroviral vectors to transfer genetic information to express new T-cell receptors that can identify a specific antigen on tumor cells while concurrently activating T-cells. These cells are then allowed to multiply in vitro (~14 days) and are eventually infused into lymphodepleted patients. These CAR-T cells grow and multiply in the patient and function to identify and perform targeted destruction of the tumor cells. The treatment is specific to the cancer cells with amplified T-cell cytotoxic responses as well as nonspecific release of cytokines which contribute to sustained and durable responses. Some off-target effects such as B cell aplasia after CD19 CAR-T cell therapy may occur, and this may predispose to infection and diminish response to vaccines, including the SARS-CoV-2 vaccines [4]. In the case of allogeneic CAR-Ts, while they can be “off the shelf” from a healthy donor, they require genomic editing to avoid graft vs. host disease [5].

The patients currently deemed appropriate for CAR-T therapies often have diseases which are refractory to or have relapsed after heavy pretreatment with conventional therapies (e.g., chemotherapy, radiation therapy, or stem cell transplantation), have limited next line of treatment options, and consequently have poor clinical outcomes, with median survival of usually less than a year. In these patients, CAR-T cell therapy can help achieve sustained response as shown in various pivotal trials that led to their FDA approval. Currently, the USA FDA has approved CAR-T cell therapy with tisagenlecleucel (tisa-cel) for pediatric ALL in children and young adults up to 25 years [6,7], axicabtagene ciloleucel (axi-cel) [1] and lisocabtagene maraleucel (liso-cel) [8] for relapsed and refractory diffuse large B cell lymphoma (DLBCL), axi-cel in relapsed and refractory follicular lymphoma [9], brexucabtagene autoleucel (brexu-cel) for relapsed and refractory mantle cell lymphoma and B-cell ALL in adults [10], and idecabtagene-vicleucel (ide-cel) [11] and ciltacabtagene autoleucel (cilta-cel) for relapsed and refractory multiple myeloma, both recognizing the B-cell maturation antigen (BCMA).

Emergence of this form of adoptive immunotherapy has resulted in improved response rates in relapsed and refractory B-cell malignancies, but at the same time, it presents unique toxicities [1,2,3]. With emphasis on cytokine release syndrome (CRS), macrophage activation syndromes (MAS), and immune effector cell associated neurotoxicity syndrome (ICANS), understanding the pathophysiology of these side-effects has been extensively examined but is still incompletely understood. While these can sometimes be life-threatening, they usually present early in the course of treatment, are well studied, and thus with appropriate management can be mitigated, thus making CAR-T cell therapy a safe treatment modality with low mortality rates [1,8,11]. With long term follow up of these patients, hematotoxicity (anemia, neutropenia, and/or thrombocytopenia) has emerged as an adverse effect of CAR-T cell therapy. This late recovery of cell counts has been largely under-recognized and under-reported, as most of the pivotal trials of CAR-T cell therapies focused on the survival benefits and acute toxicities and early cytopenia related to the lymphodepletion therapy; often cyclophosphamide and fludarabine [9,12,13].

Other immune modulating therapies have previously been shown to be associated with late-onset neutropenia, especially anti-CD20 antibodies such as rituximab [14,15,16,17]. In this case, the neutropenia is thought to be due to aberrant B-cell reconstitution and possible formation of anti-neutrophil or anti-neutrophil precursor autoantibodies [17,18]. It often occurs two to six months after therapy and can be associated with neutropenic fever. There is also evidence for growth factor expression alterations such as in stromal derived factor-1 and BAFF (B cell activating factor) which may also alter myelopoiesis after rituximab [18], and late onset neutropenia seems more associated with various Fc receptor polymorphisms [19]. The incidence of late onset neutropenia is similar after obinutuzumab as compared to after rituximab [20]. Rituximab-induced neutropenia is usually self-limited and responds to granulocyte colony stimulating factor (G-CSF) [15]. As CAR-Ts are also B-cell depleting, it is uncertain if some of the mechanisms leading to neutropenia might be similar to those operative in the case of rituximab. In the case of neutropenia after autologous stem cell transplantation, in the absence of poor engraftment due to inadequate stem cell numbers, late onset neutropenia is rare, but has often been associated with rituximab used prior to the conditioning chemotherapy as well [21]. In those who have received CAR-T therapy, the cumulative effect of prior anti-CD20 therapy on persistent cytopenia after Day+90 was not found to be statistically significant in a case series where the number of rituximab doses ranged from 2 to 19 [22].

## 2. Characteristics of Cytopenia and Findings from Various Studies

Cytopenias after infusion of CAR-T cells are exceedingly common and are biphasic or even triphasic in nature, the first phase occurring early within 3–4 weeks. In most cases [23,24], this early cytopenia is attributed to the lymphodepletion regimens, bridging chemotherapy or radiotherapy before CAR-T infusion, severe CRS, or MAS. These toxicities are responsible for an initial acute phase of the cytopenia and are managed with transfusion support, steroids, and tocilizumab, an anti-IL-6 antibody.

Prolonged or recurrent cytopenias have been increasingly reported at varying rates. In a long term follow up study of patients who had received CAR-T therapies with ongoing complete responses, sixteen percent experienced prolonged cytopenia in the absence of myelodysplasia (MDS) [25]. In a study from Hockings et al. [23] with axi-cel in 38 non-Hodgkin lymphoma (NHL) patients (with 28 DLBCL, 14 transformed follicular lymphoma, and 1 primary mediastinal B-cell lymphoma), persistent grade 3/4 neutropenia by the Common Terminology Criteria of Adverse Events (CTCAE) at day 28 after CAR-T infusion was reported in 43% of patients. This was more common in patients who had received greater than or equal to four previous lines of therapy and was independent of CRS severity. Similar findings were noted in 38 patients (ALL and NHL) treated with CD-19 directed CAR-T therapy with neutropenia noted in 62%, thrombocytopenia in 44%, and anemia in 17% of patients at 6 weeks post CAR-T cell infusion [25]. Similarly, in the recent post hoc analysis of ZUMA-1 (NCT02348216, N = 24) and ZUMA-9 (NCT03153462, N = 7) [26], using axicabtagene ciloleucel for relapsed-refractory large B cell lymphoma, grade 3–4 cytopenias at day 30 were observed in 48% of patients (29% neutropenia, 16% anemia, and 42% thrombocytopenia), with persistent grade 3–4 cytopenia in 27% at 1 year and 11% at 2 years. In this study, four patients were diagnosed with MDS after a median of 13.5 months (range 4–26 months), attributed to previous systemic therapies. In the TRANSCEND NHL-001 (N = 269) study, which evaluated the role of lisocabtagene maraleucel in relapsed-refractory large B-cell lymphoma, prolonged cytopenia (defined as ≥ grade 3, not resolved at day 29) was reported in 37% of patients [8]. In a study of 83 patients treated with axi-cel or tisa-cel or BCMA directed CAR T-cells for myeloma, cell recovery at one month was 61%, 51%, 33%, and 28% for hemoglobin, platelets, neutrophils, and WBCs, respectively. At 3 months, similar rates of count recovery were 93%, 90%, 80%, and 59%, respectively. After adjustment for baseline cytopenia and CAR construct, CRS and ICANS of grade ≥3 was associated with absence of complete count recovery at 1 month [27]. Overall, these clinical observations cannot be explained by the myelotoxic effect of the pre-treatment conditioning regimens alone given their timing and persistence. The outcomes of various studies as related to cytopenias are summarized in Table 1.

## 3. Postulated Causes of Cytopenias

The phenomenon of delayed cytopenia after CAR-T cell therapy is poorly understood with various hypotheses proposed. Factors being considered are number of previous lines of therapies, higher median age, poor bone-marrow reserve with baseline cytopenias, severity of CRS during the acute phase of treatment, roles of various inflammatory cytokines [32], high prevalence of clonal hematopoiesis of indeterminate potential (CHIP), and high baseline lactate dehydrogenase levels, possibly correlating with tumor burden. While all the above-mentioned factors may play a role in the pathogenesis, the attempts to predict and identify those at risk of this complication have produced contradictory associations [23,25,28,29].

In an analysis evaluating the outcomes in 22 patients receiving axi-cel, the authors found thrombocytopenia (≤75,000/microL) prior to infusion and median time to maximum CRS of any grade of less than 1 day to be statistically significant for development of persistent cytopenia (defined as absolute neutrophil count or ANC <500/microL without growth factor, lasting for >6 weeks). The number of previous lines of therapy was statistically significant as a contributing variable [28].

In another analysis, with aim to identify predictive biomarkers for hematotoxicity and neutropenia at day+60 as the primary end point, Rejeski [29] and colleagues in a multicenter, retrospective, real-world analysis looked into 258 patients receiving axi-cel or tisa-cel for relapsed-refractory large B-cell lymphoma and found a positive correlation between baseline thrombocytopenia and hyperferritinemia and day+60 cytopenia. Based on their findings they developed the CAR-HEMATOTOX model, which included markers associated with hematopoietic reserve (i.e., hemoglobin, platelet count, absolute neutrophil count) and baseline inflammatory markers (C-reactive protein, and ferritin). Per their analysis, high CAR-HEMATOTOX scores resulted in a longer duration of neutropenia and a higher incidence of severe thrombocytopenia and anemia. Incidence and severity of CRS and ICANS, peak cytokine levels, and number of previous lines of treatment were not associated with prolonged cytopenia. They also used three clinical phenotypes to define neutrophil recovery, (1) quick recovery: sustained neutrophil recovery without a second dip below ANC < 1000 cells/microL; (2) intermittent recovery: neutrophil recovery with ANC > 1000 cells/microL followed by second dip with ANC < 1000 cells/microL after day+21; or (3) aplastic: severe neutropenia (ANC < 500 cells/microL) for ≥14 days). In their analysis, intermittent recovery was seen in about 50% of cases, whereas 25% developed quick recovery and 25% aplastic phenotype. This model was externally validated in two different independent patient cohorts in Europe and United States with sensitivity of 89% and specificity of 68% for prediction of severe neutropenia more than or less than 14 days. The authors identified the retrospective nature and heterogeneous cohorts with small sample size as the limitations of the study [29].

CRS is contributed to by the CAR-T cells themselves, by bystander cells such as macrophages, and possibly by other cells of the tumor microenvironment. CRS is more severe with higher numbers of infused T-cells, higher tumor burden, and with use of CD28 as a co-stimulatory domain vs. those with 4-1BB constructs [33]. Myeloid-derived macrophages play a role in cytokine release syndrome through secretion of interleukin (IL)-1, IL-6 and interferon-gamma IFN-γ. Macrophage activation also occurs in response to the CAR-T infusion [34]. Toll-like receptors and downstream nuclear factor kappa-light chain enhancer of activated B cells (NF-KB) are also involved. Thus, interleukins chemokines, interferons, angiogenesis factors, and others are all implicated in this syndrome, and many of these are myelosuppressive and can contribute to both early and later cytopenias [33].

- Role of CHIP (clonal hematopoiesis of indeterminate potential) in cytopenias

The effect of CHIP on clinical outcomes in patients undergoing CAR-T cell therapy was evaluated in a recent retrospective analysis of 154 patients with NHL and MM, with median age of the population at 63 years (range 24–83 years) and median number of prior lines of treatment at four. The presence of CHIP led to an improved clinical response in the younger population <60 years of age defined as higher likelihood to achieve complete response; 77.6% with CHIP vs. 57.9% without CHIP, *p* < 0.05%). CHIP was associated with higher grade (≥2) CRS [35]. This finding contrasts with the inferior outcomes conventionally reported in patients with CHIP and NHL or MM undergoing autologous transplantation [36,37]. It was postulated that CHIP may influence the inflammasome, and CHIP had no bearing on progression free and overall survival in either older or younger patients.

- Role of cytokines in cytopenias

The role of cytokines in the development of prolonged cytopenias (beyond D+30) post-CAR-T infusion is controversial. In one study, an association between day 7 peak levels of INF-Ÿ, G-CSF, CXCL-1, IL-1β, CCL-2, IL-3, IL-6, IL-8, IL-10, and *fms*-like tyrosine (FLT-3) ligand and grade 3–4 cytopenias at day-30 after infusion was noted, and there was a correlation with low levels of epithelial growth factor (EGF) [24], emphasizing the effect of CAR-T cell activity rather than the myelosuppressive effect of conditioning regimens utilized. Low levels of stromal derived factor-1 (SDF-1), a chemokine responsible for B-cell development and trafficking of neutrophils as well as hematopoietic stem cells, were found to be associated with late onset cytopenia (after D+21) [25]. Wang et al. [30] found a 70% incidence of neutropenia, 53% severe anemia, and 48% severe thrombocytopenia in an analysis of 76 ALL patients. Neutropenia was associated with D-dimer levels and delayed peak time of CRS, anemia with delayed CRS recovery and elevated IL-10 levels, and maximum ferritin level was associated with thrombocytopenia. In general, a higher grade of CRS was associated with prolonged cytopenias. In another retrospective analysis of 173 patients, 9% had persistent neutropenia (D+28 post CAR-T infusion) and 14% prolonged thrombocytopenia after a CD19-targeted CAR-T construct utilized in a single institution study [31]. CRS severity was an independent variable for decreased platelet count, and lower pre-lymphodepletion platelet count was an independent predictor of both platelet count and neutrophil count suppression. In multivariable analysis, higher IL-6 levels were associated with lower day 28 counts, and higher concentrations of transforming growth factor (TGF)-beta were associated with higher counts [31]. As hematologic toxicity seems to be a class effect seen with most CAR constructs, there is also an ongoing discussion if this is secondary to the expansion and persistence of CAR-T cells themselves [23] or could be due to the different costimulatory domains (CD-28 for axi-cel and brexu-cel and 4-1BB for tisa-cel and liso-cel) [38]. Other analyses have not found an association between CRS incidence and severity nor with peak cytokine levels and neutropenia to day+60 [28].

## 4. Management of CAR-T Induced Cytopenias

Management of this clinical entity remains largely symptomatic with use of transfusion products and supportive care with colony stimulating factors. Use of the validated CAR-HEMATOTOX model can help in risk stratification for predicting early and late hematotoxicity [29]. Early initiation of prophylactic G-CSF [39] in high-risk patients after CRS has subsided, along with appropriate antiviral and antifungal prophylaxis, and close monitoring for any infectious complications are important. The use of granulocyte/macrophage growth factor in particular is not recommended for the first 2 to 3 weeks of therapy given concerns about exacerbating CRS. The possible role of inflammatory mediators in contributing to cytopenia has led some to recommend a trial of steroids for mitigation [40], and some have advocated use of anti-cytokine therapy such as anakinra or tocilizumab, although a role for these in prolonged cytopenias is uncertain. Although this remains to be studied further, high-risk patients may be triaged for autologous stem cell collection and cryopreservation for hematological rescue in the case of prolonged cytopenias after CAR-T infusion [41,42]. Those who have previously collected stem cells for autologous transplantation purposes could have these cryopreserved stem cells reinfused as a rescue. There is currently no general consensus regarding safe use of granulocyte colony stimulating factor and thrombopoietin-receptor agonists and, in general, for cytopenias lasting more than a month, marrow examination is recommended, as this can rule out involvement with the primary malignancy or with myelodysplastic syndrome/acute myelogenous leukemia as a cause of cytopenias [43]. While prophylaxis against viruses and *Pneumocytis jurovecii* pneumonia (PJP) are recommended, there is no consensus about anti-bacterial prophylaxis or anti-fungal prophylaxis in late onset cytopenia cases [40,43]. The European Society for Blood and Marrow Transplantation has now suggested antiviral and PJP prophylaxis from lymphodepletion until 1 year post-CAR-T infusion and until CD4 counts are >0.2 × 10^9^/L. Granulocyte colony stimulating factors are to be avoided during early phases during times of increased risk of CRS and ICANS, but they can be used after Day+14 on an individualized basis. Anti-bacterial prophylaxis is per institutional guidelines, and intravenous immunoglobulin can be considered in adults with serious or recurrent infections and IgG levels <400 mg/dL [44].

## 5. Conclusions

**Figure 1:**
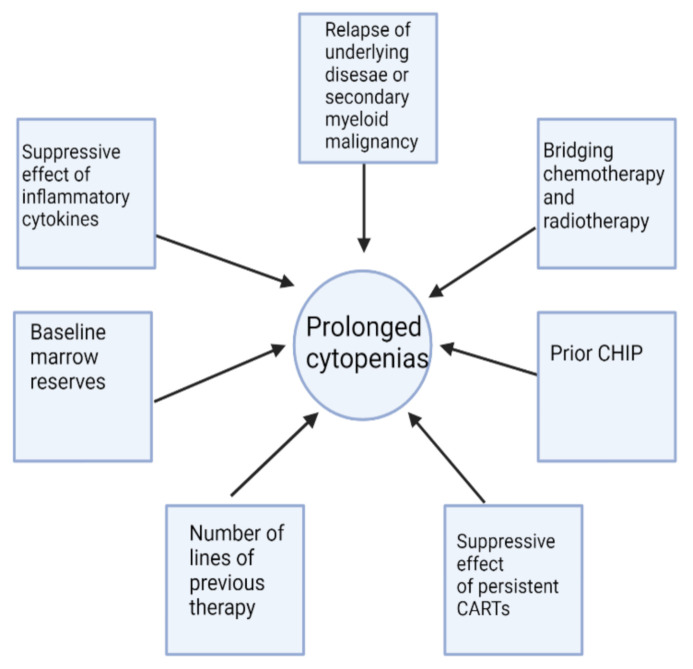
Some possible factors contributing to cytopenias in CAR-T therapy. Several of these variables may be active concurrently. While bridging chemotherapy and radiotherapy contribute to early cytopenias, their occurrence may also influence late cytopenias. Figure generated in Biorender.com.

Cytopenias of grade 3 or higher and lasting more than one month after CAR T-cell infusion occur in 20–40% of patients [44]. In a series of “real-life” CAR-T therapy, cytopenia beyond 90 days was found in 33% of evaluable patients [45]. In the ZUMA-7 trial, where axi-cel was compared to high dose therapy and stem cell rescue, 29% of patients had prolonged cytopenias of grade 3 or higher after 30 days in the axi-cel arm compared with only 19% who received high-dose chemotherapy [46]. As it becomes increasingly recognized, late cytopenia after CAR-T cell therapy poses a clinical challenge in risk stratification, diagnosis, and management. The reporting of this late side effect has been inconsistent. The definition of prolonged/recurrent cytopenia is heterogeneous and arbitrary, with wide time ranges from D+14 up to D+90 being used as a framework for examination of the incidence of cytopenia. The mechanisms for these count depressions remain poorly understood with multiple proposed hypotheses and associated factors that have not been consistent among various predictive models, thereby making it a moving target for any clinical intervention. Figure 1 demonstrates some possible contributors to cytopenias post-CAR-T therapy. Table 2 illustrates an arbitrary breakdown of the timeframe of cytopenias with postulated mechanisms and proposed interventions. As noted, future work will be required to understand the incidence, causation, and ramifications of cytopenias at these various time points on the ultimate effectiveness of CAR-T therapies. It is also important to remember that cytopenias can be early and self-limited, intermittent, continuous, or with de novo late appearance [29].

Cytopenias can negatively impact CAR-T therapy outcomes with increased infectious complications and increased utilization of medical resources, thereby adding cost to an already expensive treatment [47]. However, other studies have shown that with the exception of neutropenia, increase in duration of anemia or thrombocytopenia may be associated with improvement in progression-free survival. There is evidence that cytopenias after CAR-T therapy may not be associated with an effect on risk of disease relapse [48]. In a retrospective analysis, Lerman and colleagues examined whether complete vs. incomplete count recovery defined as an absolute neutrophil count >1000/micoL and platelets >100,000/microL affected relapse free survival or overall survival. There was no difference in relapse free survival when stratified by hematologic recovery, but overall survival was lower for those with incomplete count recovery. In a multivariable analysis which adjusted for gender, prior blinatumomab, number of relapses, disease burden at infusion, and maximum CRS grade, complete count recovery was not associated with overall survival with a hazard ratio of 0.74 (*p* = 0.2908) as compared to incomplete count recovery [48]. In addition to increasing morbidity and mortality, given that the majority of patients ultimately experience disease relapse, cytopenias compromise the ability to provide additional treatment post-relapse and may preclude participation in clinical trials. In a series from the Mayo system of patients who relapsed after CAR-T therapy, low blood counts were the most common barrier to participation in clinical trials [49].

The American Society of Clinical Oncology has adopted guidelines which address management of post-CAR-T cytopenias and B-cell aplasia [40] as has the European Society for Blood and Marrow Transplantation [44]. The role that prior therapies, baseline blood counts, type of lymphodepleting [50] or bridging therapy, CAR-T construct utilized, severity of CRS and ICANS, persistence of CAR-Ts after infusion, and other factors play in cytopenia incidence continue to be examined. Current reported studies are primarily retrospective, heterogeneous in terms of CAR-T used and disease treated, non-uniform in definitions of cytopenias and durations for end points and vary in terms of recommended management. Prospective studies will be required in the future, and correlative studies investigating the pathophysiology of prolonged cytopenias should be incorporated into clinical trials. It is anticipated that as more CAR-T constructs achieve widespread use, the incidence, pathogenesis, and management of related cytopenias will be further elucidated with the goal of overcoming this potential late toxicity of these cellular therapies.

**Table 2:** Possible classification and management of post-CAR-T cytopenias.

Timeline	Very Early	Early	Late
Time	Up to 30 Days	Up to 90 days	>90 days
Causes	-Lymphodepleting regimens -CRS	-Delayed effects of CRS	-Multiple factors (See Figure 1)
Interventions	-Tociluzimab/Dexamethasone for CRS -Transfusion support -Avoid granulocyte colony stimulating factor in first 14–21 days -Antibiotic prophylaxis and empiric coverage for fevers -Possible alteration of lymphodepleting regimens [50]	-Possible role for anti-inflammatory agents -Granulocyte colony stimulating factor -Thrombopoietin receptor agonists -Transfusion support as needed	-Granulocyte colony stimulating Factor -Thrombopoietin receptor agonists -Transfusion support as needed -Possible immunomodulatory therapy (not yet systematically explored) -Autologous stem cell rescue if a cryopreserved product is available -Consider marrow examination

## Figures and Tables

**Table 1:** Incidence of Cytopenias In a Sampling of CAR-T Studies.

Study/Reference	Sample Size	Disease	Study	CAR-T Used	Incidence of Delayed Cytopenia
Hockings C. et al. [23]	39	DLBCL, t FL, PMBCL	Retrospective analysis	Axi-cel, Tisa-gen	At D+28 Neutropenia (grade 3–4)-43%
Cordeiro A. et al. [24]	86	R/R ALL, NHL, CLL	Retrospective analysis of phase 1–2	Locally produced CAR-T with 4-1BB co-stimulatory domain	At D+90 16% requiring transfusions, or growth factors, without MDS
Fried S. et al. [25]	39	R/R ALL, NHL	Retrospective analysis of phase 1–2	Locally produced CAR-T with CD-28 co-stimulatory domain	At D+42 Neutropenia-62% Thrombocytopenia-44% Anemia-17%
Strati P. et al. [26]	31	R/R Large B-cell Lymphoma	Retrospective analysis of ZUMA-1 and ZUMA-9	Axi-cel	At D+30 Neutropenia (grade3–4)-29% Anemia-16% Thrombocytopenia-42% (patients with ongoing remission grade 3–4 cytopenia in 11% at 2 years)
Abramson J.S. et al. [8]	269	R/R B-cell Lymphoma	Phase 1	Liso-cel	At D+29 Cytopenia (grad 3–4)-37%
Jain T. et al. [27]	83	B-cell Lymphoma, B-ALL, Multiple Myeloma	Retrospective analysis	Axi-cel, Tisa-gen, BCMA	At D+90 Neutropenia-20% Anemia-7% Thrombocytopenia-10%
Nahas G.R. et al. [28]	22	R/R B-cell lymphoma	Retrospective analysis	Axi-cel	At D+42 Cytopenia (ANC <500/microL or requiring filgastrim to maintain ANC >500)-38%
Rejeski K et al. [29]	258	R/R B-cell Lymphoma	Retrospective analysis	Axi-cel, Tisa-cel	At D+21 Neutropenia (ANC <500/microL)-64%
Wang et al. [30]	76	B-ALL	Retrospective analysis of phase 1–2	Locally produced CAR-T with 4-1BB co-stimulatory domain	At D+80 Severe Neutropenia-70% Severe anemia-53% Severe thrombocytopenia-48%
Juluri et al. [31]	173	B-ALL, NHL, CLL	Retrospective analysis of phase 1–2	Locally produced Car-T with 4-1BB co-stimulatory domain	At D+28 Neutropenia-9% Thrombocytopenia 14%

DLBCL—Diffuse Large b-cell lymphoma, t FL—transformed Follicular lymphoma, PMBCL—Primary Mediastinal B-cell lymphoma, R/R ALL—relapsed-refractory Acute Lymphoblastic lymphoma, and NHL—Non-Hodgkin lymphoma.
